# Clinical Significance of Klotho, Fibroblast Growth Factor 23, and Their Ratio for Acute Kidney Injury in Sepsis Patients

**DOI:** 10.1159/mpp/adpag028

**Published:** 2026-10-09

**Authors:** Sumian Dou, Yuchao Shen, Botao Zhao, Ling Wang, Wei Zhang, Jing Wang, Chunxue Wu, Long Yang, Zhanghai Sun, Ying Cui

**Affiliations:** a West Branch Emergency Department, Cangzhou Central Hospital, Cangzhou, Hebei, China; b Cangzhou Central Hospital, The Fifth Department of Coronary Heart Disease, Cangzhou, Hebei, China; c Department of Oral Pathology, Cangzhou Medical College, Cangzhou, Hebei, China; d Department of Mental Health Education, Cangzhou Medical College, Cangzhou, Hebei, China

**Keywords:** Sepsis-associated acute kidney injury, Klotho, Fibroblast growth factor 23, Klotho/FGF23 ratio, Discrimination

## Abstract

**Objective:**

The sepsis-associated acute kidney injury (SA-AKI) is common in patients with sepsis and associated with poor outcomes, yet serum creatinine and urine output have limited value for early assessment. We examined whether admission Klotho, fibroblast growth factor 23 (FGF23), and the Klotho/FGF23 ratio were associated with SA-AKI and short-term outcomes and compared their discriminative performance.

**Methods:**

We conducted a retrospective cohort study of 320 patients admitted to the intensive care unit (ICU) with Sepsis-3-defined sepsis. SA-AKI (*n* = 112) was retrospectively identified according to Kidney Disease: Improving Global Outcomes (KDIGO) criteria. Klotho and FGF23 were measured by enzyme-linked immunosorbent assay in stored serum samples collected within 24 h of ICU admission. Discrimination was assessed by receiver operating characteristic analysis, and adjusted associations were evaluated using multivariable logistic and linear regression.

**Results:**

Patients with SA-AKI had lower Klotho, higher FGF23, and a reduced Klotho/FGF23 ratio at admission (all *p* < 0.001). The ratio showed the highest area under the curve biomarkers for SA-AKI (0.84; 95% confidence interval 0.79–0.88) and remained associated with SA-AKI after multivariable adjustment (odds ratio 2.25; 95% confidence interval 1.46–3.53). The ratio decreased with increasing KDIGO stage. Among patients with SA-AKI, a low ratio remained associated with lower 3-month renal recovery, longer ICU and hospital stays, higher in-hospital mortality, and greater 3-month chronic kidney disease progression after adjustment.

**Conclusion:**

The admission Klotho/FGF23 ratio was associated with SA-AKI, higher KDIGO stage, and short-term outcomes and showed the highest observed discrimination among the three biomarkers.

**Highlights of the Study**

- Admission Klotho/fibroblast growth factor 23 ratio detects sepsis-associated acute kidney injury early with excellent accuracy.
- A lower ratio independently predicts sepsis-associated acute kidney injury.
- The ratio stratifies renal recovery, mortality, and chronic kidney disease progression.

## Introduction

The sepsis-associated acute kidney injury (SA-AKI) is a frequent complication in critically ill patients with sepsis. It is defined as acute kidney injury (AKI) meeting Kidney Disease: Improving Global Outcomes (KDIGO) criteria in patients who fulfill the Sepsis-3 definition of sepsis [1]. SA-AKI incidence is increasing in intensive care units (ICUs) and is associated with substantially higher mortality [2]. Marked clinical heterogeneity in presentation, progression, and treatment response further complicates management [3]. SA-AKI is associated with longer ICU and hospital stay and a higher long-term risk of chronic kidney disease (CKD) and cardiovascular events [4, 5]. Nearly half of patients who progress to KDIGO stage 3 require kidney replacement therapy, yet renal recovery remains limited [6]. Late-onset SA-AKI (≥48 h after ICU admission) carries a worse prognosis than early-onset disease, highlighting the need for earlier recognition and intervention [7].

The diagnosis of SA-AKI is based on serum creatinine (Scr) and urine output, but both are limited. Scr rises late, typically 24–72 h after injury. Urine output is affected by volume status and diuretics [8]. The 28th Acute Disease Quality Initiative recommends integrating functional and injury biomarkers to improve early assessment [1, 5]. Emerging evidence suggests that fibroblast growth factor 23 (FGF23) and Klotho may be informative in this setting [4, 8], with FGF23 increasing rapidly after AKI while Klotho declines early [9, 10]. Klotho is predominantly produced by the kidney and exerts anti-inflammatory, antioxidant, and endothelial-protective effects [10, 11]. In SA-AKI, renal Klotho expression decreases with injury severity. In contrast, the bone-derived hormone FGF23 rises early and may aggravate injury by amplifying inflammation and disrupting calcium-phosphate homeostasis [9, 12]. Therefore, imbalance of the FGF23-Klotho axis may contribute to SA-AKI, with reduced Klotho signaling and excessive FGF23 activity promoting cardiorenal injury [11–13]. Previous studies have evaluated Klotho and FGF23 individually or as components of multimarker approaches in patients with sepsis, AKI, and critical illness. Pei et al. [8] measured Klotho and FGF23 together with cystatin C, kidney injury molecule-1, and neutrophil gelatinase-associated lipocalin in a prospective cohort of patients with sepsis, whereas Toro et al. [4] evaluated a combined indicator incorporating FGF23, Klotho, and erythropoietin in critically ill patients with sepsis. Clinical and experimental studies have also demonstrated reduced renal Klotho expression during sepsis-associated kidney injury [10], and reviews have summarized the reciprocal increase in FGF23 and decrease in Klotho during AKI [9]. In addition, elevated FGF23 has been associated with AKI, mortality, and other adverse outcomes in critically ill patients [14]. The Klotho/FGF23 ratio has been associated with renal disease progression in patients with CKD [15], but it has been less well characterized in SA-AKI. In particular, it remains unclear whether the ratio shows greater discrimination for SA-AKI than either biomarker alone and whether it is associated with SA-AKI severity and short-term clinical outcomes.

In the light of above mentioned reports, we conducted an exploratory retrospective cohort study of 320 patients with sepsis to (i) identify clinical and biomarker factors independently associated with SA-AKI; (ii) compare the discriminative performance of Klotho, FGF23, and the Klotho/FGF23 ratio; (iii) assess their associations with SA-AKI severity; and (iv) examine the association of the Klotho/FGF23 ratio with short-term clinical outcomes.

## Materials and Methods

### Participants

This single-center retrospective cohort study included patients with sepsis admitted to the general ICU of Cangzhou Central Hospital between January 2021 and December 2024. Clinical data were retrospectively extracted from the hospital electronic medical record system, whereas serum samples used for biomarker measurements were retrospectively obtained from the hospital central biobank. The study was approved by Cangzhou Central Hospital (#2025-173-01), and informed written consent was waived.

### Study Design and Data Sources

Baseline clinical characteristics at admission were compared between patients with SA-AKI and those without SA-AKI. Variables included demographics, infection characteristics, comorbidities, severity scores (Sequential Organ Failure Assessment [SOFA] and Acute Physiology and Chronic Health Evaluation II [APACHE II]), and laboratory indices (inflammatory markers, renal function markers, serum Klotho, and FGF23). Univariate analyses were performed to screen factors associated with SA-AKI occurrence. The estimated glomerular filtration rate (eGFR) was calculated using the 2009 Scr-based equation [16].

### Clinical Data Collection

Demographic characteristics, infection sites, comorbidities, laboratory findings, severity scores, and clinical outcomes were obtained from the electronic medical record system. Urine output was measured hourly through indwelling urinary catheters and recorded in the nursing records. Exposure to nephrotoxic drugs within 72 h after ICU admission and vasopressor use within 24 h after ICU admission were recorded. The timing and modality of kidney replacement therapy initiated after ICU admission were also collected.

### Eligibility Criteria

Inclusion criteria were as follows: (1) age ≥18 years; (2) sepsis defined by Sepsis-3 criteria, i.e., infection with a SOFA score ≥2; (3) complete clinical data, including baseline characteristics, laboratory results, treatment information, and outcome data; (4) ICU length of stay ≥24 h; and (5) availability of a serum sample at admission.

Exclusion criteria were as follows: (1) pre-existing chronic kidney disease [17], prior kidney replacement therapy (hemodialysis or peritoneal dialysis), or history of kidney transplantation; (2) other severe organ dysfunction, including acute myocardial infarction or severe hepatic failure; (3) advanced malignancy or patients receiving hospice care; and (4) incomplete clinical data with missing key variables.

### Definition and Ascertainment of SA-AKI

Patients were retrospectively classified into the SA-AKI group (*n* = 112) and the non-SA-AKI group (*n* = 208) according to whether they met the KDIGO criteria for AKI within 7 days after the diagnosis of sepsis. At the time of serum sampling, SA-AKI status had not been established. SA-AKI was diagnosed according to the KDIGO 2012 criteria: an increase in Scr ≥26.5 μmol/L within 48 h, or an increase in Scr ≥50% from baseline within 7 days, or urine output <0.5 mL/(kg·h) for ≥6 h. Urinary tract obstruction and other non-renal causes of reduced urine output were excluded. Baseline Scr was defined as the most recent outpatient or inpatient Scr value obtained within 3 months before admission. For patients without an available preadmission Scr measurement (*n* = 47, 14.7%), the lowest Scr value measured within 24 h after admission was used as the baseline. Urine output was measured hourly through an indwelling urinary catheter and obtained from the nursing records.

### Acute Kidney Injury Staging

AKI stages were defined using KDIGO 2012 criteria [18]:

Stage 1: Scr increase ≥26.5 μmol/L or 1.5–1.9× baseline; or urine output <0.5 mL/(kg·h) for 6–12 h.
Stage 2: Scr increase 2.0–2.9× baseline; or urine output <0.5 mL/(kg·h) for ≥12 h.
Stage 3: Scr increase ≥3.0× baseline or Scr ≥353.6 μmol/L (≥4.0 mg/dL); or urine output <0.3 mL/(kg·h) for ≥24 h; or initiation of kidney replacement therapy. The timing and modality of kidney replacement therapy were recorded, and its initiation was considered a criterion for KDIGO stage 3.

### Biomarker Measurement

Serum Klotho and FGF23 were not routinely measured at ICU admission. Residual serum samples from peripheral venous blood collected within 24 h of ICU admission for routine clinical testing were retrieved from the hospital central biobank. Blood samples were centrifuged at 3,000 rpm for 10 min at 4°C within 30 min of collection. The serum was aliquoted into 200 µL cryovials and immediately stored at −80°C until analysis. All samples were analyzed without repeated freeze-thaw cycles. Serum Klotho was quantified using an enzyme-linked immunosorbent assay (ELISA) kit (EH2184; Wuhan Fine Biotech, Wuhan, China) according to the manufacturer’s instructions. This kit detects soluble human alpha-Klotho, representing the full-length extracellular domain, but not membrane-bound Klotho or beta-Klotho. Serum FGF23 was quantified using a separate ELISA kit (EH3058; Wuhan Fine Biotech, Wuhan, China) according to the manufacturer’s instructions. This kit detects C-terminal FGF23, including both intact FGF23 and C-terminal fragments, but not N-terminal fragments.

### SOFA Scoring

The SOFA score was used to assess dysfunction in six organ systems (respiratory, cardiovascular, hepatic, coagulation, central nervous system, and renal), with each component scored from 0 to 4 (total score 0–24) [19]. The highest SOFA score within 24 h after admission was used.

### APACHE II Score

The APACHE II score includes an acute physiology score (12 physiologic variables), an age component, and a chronic health component (range, 0–71) [20], with higher scores indicating greater disease severity. The APACHE II score was calculated within 24 h of admission.

### Statistical Analysis

Continuous variables are presented as mean ± standard deviation (SD) or median (interquartile range [IQR]), depending on normality. Categorical variables are presented as *n* (%). Between-group comparisons (SA-AKI vs. non-SA-AKI) were performed using an unpaired *t* test with Welch’s correction for normally distributed variables, or the Mann-Whitney U test otherwise. Categorical variables were compared using Fisher’s exact test. All tests were two-sided, and *p* < 0.05 was considered statistically significant. Receiver operating characteristic curves were used to assess the discriminative performance of admission Klotho, FGF23, and the Klotho/FGF23 ratio. The area under the curve (AUC) with 95% confidence interval (CI) was reported. Optimal cut-offs were determined by the maximum Youden index (sensitivity + specificity − 1), with corresponding sensitivity and specificity. Variables with *p* < 0.05 in univariate analyses were considered for multivariable logistic regression. Multicollinearity was assessed using the variance inflation factor (VIF), with values >5 indicating substantial collinearity. When collinearity was identified, the less clinically relevant or less specific variable was excluded. Variables independently associated with SA-AKI were selected using forward stepwise logistic regression based on the likelihood ratio test. As all included patients had complete baseline, laboratory, and outcome data, no missing-data imputation was performed. Model calibration was assessed using the Hosmer-Lemeshow goodness-of-fit test. Internal validation was performed using 1,000 bootstrap resamples, and the optimism-corrected AUC and 95% CI were calculated.

## Results

### Univariate Analysis of Admission Characteristics Associated with SA-AKI Occurrence

To screen admission variables associated with SA-AKI and to inform subsequent multivariable and biomarker analyses, baseline admission characteristics were compared between patients with and without SA-AKI (Table 1). Older age and a higher prevalence of coronary heart disease were observed in the SA-AKI group, whereas sex, infection site, hypertension, diabetes mellitus, and hyperlipidemia did not differ between the groups. Patients with SA-AKI had higher SOFA and APACHE II scores, arterial lactate, C-reactive protein (CRP), and procalcitonin (PCT) in SA-AKI. Admission Scr and eGFR were not significantly different. Patients with SA-AKI also had lower Klotho, higher FGF23, and a reduced Klotho/FGF23 ratio (all *p* < 0.001). Variables showing between-group differences were entered into multivariable models.

**Table 1.** Analysis of Single Factors at Admission That Affected the Occurrence of Acute Kidney Injury in Sepsis Patients

Factors	NAKI (*n* = 208)	SA-AKI (*n* = 112)	*p* value
Age (years), median (IQR)	63 (50–74)	68 (56–80)	0.007
Male gender, *n* (%)	117 (56.3)	67 (59.8)	0.555
Primary infection site, *n* (%)			
Respiratory system	61 (29.3)	24 (21.4)	0.193
Abdomen	109 (52.4)	65 (58.0)	
Blood	17 (8.2)	15 (13.4)	
Other	21 (10.1)	8 (7.2)	
Hypertension, *n* (%)	81 (38.9)	46 (41.1)	0.721
Diabetes mellitus, *n* (%)	53 (25.5)	33 (29.5)	0.509
Coronary heart disease, *n* (%)	16 (7.7)	20 (17.9)	0.009
Hyperlipidemia, *n* (%)	54 (25.9)	35 (31.3)	0.360
SOFA score (points), median (IQR)	4 (3–6)	6 (4–9)	0.001
APACHE II score (points), median (IQR)	14 (11–17)	19 (16–23)	<0.001
Arterial lactate (mmol/L), median (IQR)	1.9 (1.3–2.7)	2.7 (1.8–5.2)	<0.001
Scr (μmol/L), mean±SD	86.43±15.36	90.05±16.41	0.152
eGFR (mL/min/1.73 m^2^), mean±SD	87.23±14.71	83.18±14.29	0.206
CRP (mg/L), median (IQR)	113.51 (84.17–220.63)	164.78 (112.45–241.57)	0.003
PCT (ng/mL), median (IQR)	1.22 (0.21–4.34)	4.36 (1.52–22.06)	<0.001
Klotho (ng/mL), mean±SD	540.67±154.91	384.04±139.14	<0.001
FGF23 (pg/mL), mean±SD	79.54±22.86	102.61±27.64	<0.001
Klotho/FGF23 ratio, median (IQR)	7.24 (5.39–9.23)	3.75 (2.78–4.99)	<0.001

The data were shown as mean ± SD or median (interquartile range) or *n* (percentage). The comparisons of data between the two groups were done by Mann-Whitney test or Unpaired *t* test with Welch’s correction or Fisher’s exact test.

SA-AKI, sepsis-associated acute kidney injury; SOFA, Sequential Organ Failure Assessment; APACHE II, Acute Physiology and Chronic Health Evaluation II; eGFR, estimated glomerular filtration rate.

### Altered Klotho-FGF23 Profile at Admission among Patients with SA-AKI

To compare the admission Klotho-FGF23 profile between groups, serum Klotho, FGF23, and the Klotho/FGF23 ratio were compared between the SA-AKI and non–SA-AKI groups (Fig. 1). At admission, lower Klotho concentrations (384.04 ± 139.14 vs. 540.67 ± 154.91 pg/mL; Fig. 1a), higher FGF23 concentrations (102.61 ± 27.64 vs. 79.54 ± 22.86 pg/mL; Fig. 1b), and a reduced Klotho/FGF23 ratio (median 3.75 vs. 7.24; Fig. 1c) were observed in the SA-AKI group. These findings indicate an unfavorable admission Klotho-FGF23 profile in patients with SA-AKI.

**Fig. 1.**
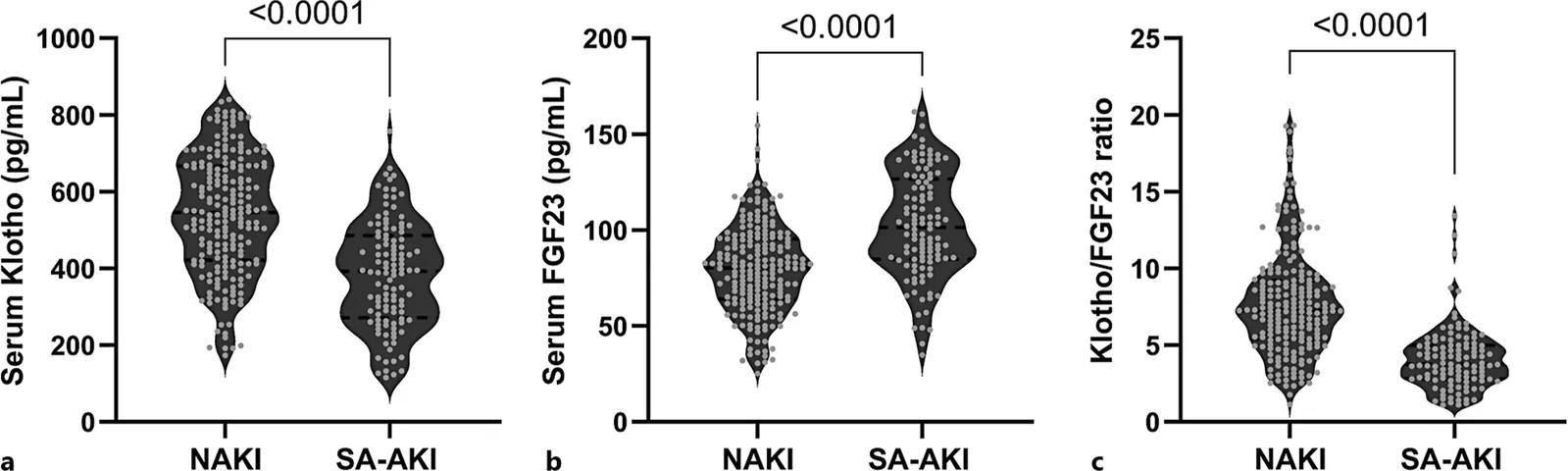
Comparison of serum Klotho (**a**), FGF23 levels (**b**), and Klotho/FGF23 ratio (**c**) at admission between SA-AKI and NAKI (non–SA-AKI). Measurement data conforming to normal distribution were compared by independent samples *t* test; data not conforming to normal distribution were compared by Mann-Whitney *U* test.

### Correlations of Admission Klotho, FGF23, and the Klotho/FGF23 Ratio with Renal Function and Inflammatory Markers

Correlation analyses were performed to explore the associations of admission Klotho, FGF23, and the Klotho/FGF23 ratio with renal function and systemic inflammation (online suppl. Fig. S1; for all online suppl. material, see https://doi.org/10.1159/mpp/adpag028). Klotho was positively correlated with eGFR (*r* = 0.36, online suppl. Fig. S1b) and negatively correlated with FGF23 (*r* = −0.52, online suppl. Fig. S1a), CRP (*r* = −0.40, online suppl. Fig. S1c), and PCT (*r* = −0.35, online suppl. Fig. S1d). FGF23 was negatively correlated with eGFR (*r* = −0.38, online suppl. Fig. S1e) and positively correlated with CRP (*r* = 0.35, online suppl. Fig. S1f) and PCT (*r* = 0.29, online suppl. Fig. S1g). The Klotho/FGF23 ratio was positively correlated with eGFR (*r* = 0.43, online suppl. Fig. S1h) and negatively correlated with CRP (*r* = −0.42, online suppl. Fig. S1i) and PCT (*r* = −0.45, online suppl. Fig. S1j). All correlations were statistically significant (all *p* < 0.001). These findings suggest that lower Klotho, higher FGF23, and a lower Klotho/FGF23 ratio were associated with lower eGFR and greater systemic inflammation.

### Discriminative Performance of Admission Klotho, FGF23, and the Klotho/FGF23 Ratio for SA-AKI

To quantify discrimination for SA-AKI, receiver operating characteristic analyses were performed for admission Klotho, FGF23, and the Klotho/FGF23 ratio (Fig. 2; Table 2). The Klotho/FGF23 ratio had the highest AUC of 0.84 (95% CI, 0.79–0.88; *p* < 0.001). An optimal threshold of 5.49 was identified, yielding 83.93% sensitivity and 73.56% specificity. Lower AUCs were observed for Klotho (0.77; 95% CI, 0.72–0.82) and FGF23 (0.74; 95% CI, 0.68–0.80). Using their respective optimal cut-offs (460.52 pg/mL for Klotho; 84.76 pg/mL for FGF23), sensitivities of 70.54% and 75.00% and specificities of 69.23% and 60.58% were obtained. Overall, the Klotho/FGF23 ratio had the highest observed AUC among the three biomarkers.

**Fig. 2.**
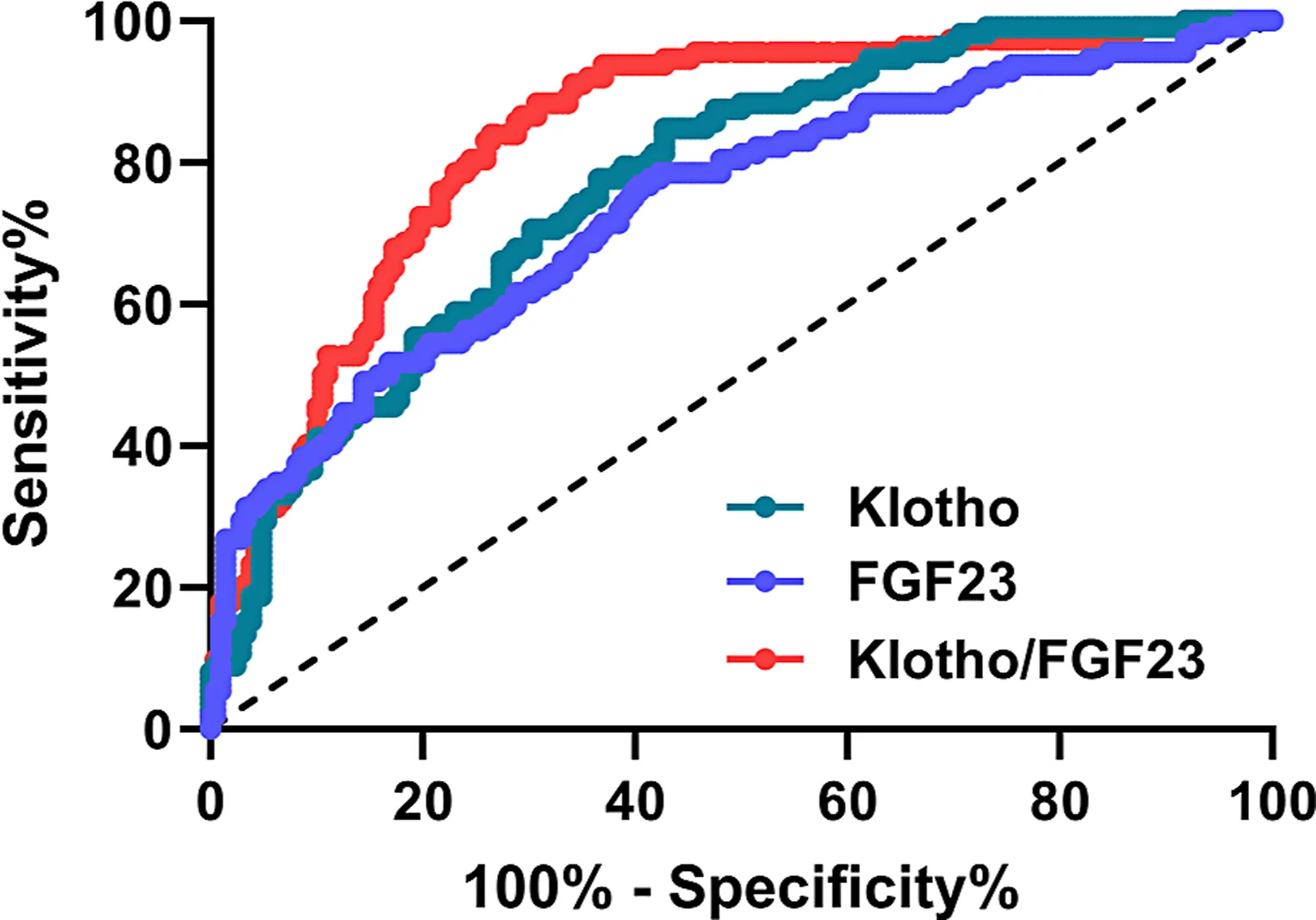
ROC curves showing the discrimination of SA-AKI by admission Klotho, FGF23, and the Klotho/FGF23 ratio. ROC, receiver operating characteristic.

**Table 2.** Predictive Values of Klotho, FGF23, and Klotho/FGF23 Ratio for SA-AKI

Biomarkers	AUROC (95% CI)	Cut-off value	Sensitivity (%)	Specificity (%)	*p* value
Klotho	0.77 (0.72–0.82)	460.52 pg/mL	70.54	69.23	<0.001
FGF23	0.74 (0.68–0.80)	84.76 pg/mL	75.00	60.58	<0.001
Klotho/FGF23 ratio	0.84 (0.79–0.88)	5.49	83.93	73.56	<0.001

AUROC, area under the receiver operating characteristic curve; CI, confidence interval.

### Factors Independently Associated with SA-AKI in Multivariable Logistic Regression

Multivariable logistic regression was performed to identify factors independently associated with SA-AKI (Table 3). Variables with *p* < 0.10 in univariate analyses were entered as candidates. Because collinearity was observed between APACHE II and SOFA (VIF = 8.7; *r* = 0.79) and between CRP and PCT (VIF = 7.3; *r* = 0.76), SOFA and PCT were retained, whereas APACHE II and CRP were excluded. The final model included age ≥65 years, coronary heart disease, SOFA score, arterial lactate, PCT, FGF23 >84.76 pg/mL, Klotho <460.52 pg/mL, and a Klotho/FGF23 ratio <5.49.

**Table 3.** Independent Risk Factors for SA-AKI (Multivariate Logistic Regression Analysis)

Risk factors	Odds ratio (95% CI)	*p* value
Age ≥65 years	1.64 (1.17–2.85)	0.012
Coronary heart disease	1.47 (0.89–2.08)	0.184
SOFA score (per 1-point increase)	1.52 (1.28–1.81)	<0.001
Arterial lactate (per 1 mmol/L increase)	1.33 (1.07–1.59)	0.005
PCT (per 1 ng/mL increase)	1.26 (1.09–1.47)	0.008
FGF23 >84.76 pg/mL	1.39 (1.05–1.72)	0.021
Klotho <460.52 pg/mL	1.62 (1.17–2.31)	0.006
Klotho/FGF23 ratio <5.49	2.25 (1.46–3.53)	<0.001

SA-AKI, sepsis-associated acute kidney injury; SOFA, Sequential Organ Failure Assessment; FGF23, fibroblast growth factor 23; CI, confidence interval.

In adjusted analyses, a Klotho/FGF23 ratio <5.49 was associated with the highest odds of SA-AKI (odds ratio [OR] 2.25; 95% CI, 1.46–3.53; *p* < 0.001). Independent associations were also observed for low Klotho (OR 1.62; 95% CI, 1.17–2.31; *p* = 0.006) and elevated FGF23 (OR 1.39; 95% CI, 1.05–1.72; *p* = 0.021). Higher SOFA score (per point: OR 1.52; 95% CI, 1.28–1.81; *p* < 0.001), arterial lactate (per 1 mmol/L: OR 1.33; 95% CI, 1.07–1.59; *p* = 0.005), PCT (per 1 ng/mL: OR 1.26; 95% CI, 1.09–1.47; *p* = 0.008), and age ≥65 years (OR 1.64; 95% CI, 1.17–2.85; *p* = 0.012) were also independently associated with SA-AKI, whereas coronary heart disease was not (*p* = 0.184).

The Hosmer-Lemeshow test showed no evidence of poor model fit (*p* = 0.68). The optimism-corrected AUC after 1,000 bootstrap resamples was 0.82 (95% CI, 0.77–0.87), close to the original AUC of 0.84, indicating good model stability and limited optimism. Overall, the Klotho/FGF23 ratio remained independently associated with SA-AKI after adjustment for illness severity, metabolic stress, and systemic inflammation, supporting further evaluation in prospective studies.

### Klotho Decreases and FGF23 Increases with Higher KDIGO Stage in SA-AKI

To determine whether admission biomarkers tracked SA-AKI severity, serum Klotho, FGF23, and the Klotho/FGF23 ratio were compared across KDIGO stages (Fig. 3). Klotho declined with increasing stage (444.3 ± 131.9, 376.3 ± 115.3, and 281.2 ± 123.2 pg/mL; overall *p* < 0.0001), with significant differences between adjacent stages (Fig. 3a). In contrast, FGF23 increased stepwise (89.01 ± 25.59, 105.2 ± 21.27, and 124.5 ± 24.79 pg/mL; overall *p* < 0.0001; stage 1 vs. 2, *p* = 0.0059; stage 2 vs. 3, *p* = 0.0066) (Fig. 3b). The Klotho/FGF23 ratio also decreased progressively (4.973 ± 2.433, 3.833 ± 1.268, and 2.806 ± 1.391; overall *p* < 0.0001; stage 1 vs. 2, *p* = 0.0186; stage 2 vs. 3, *p* = 0.0127) (Fig. 3c). These findings indicate that a more unfavorable Klotho-FGF23 profile at admission was associated with higher KDIGO stage.

**Fig. 3.**
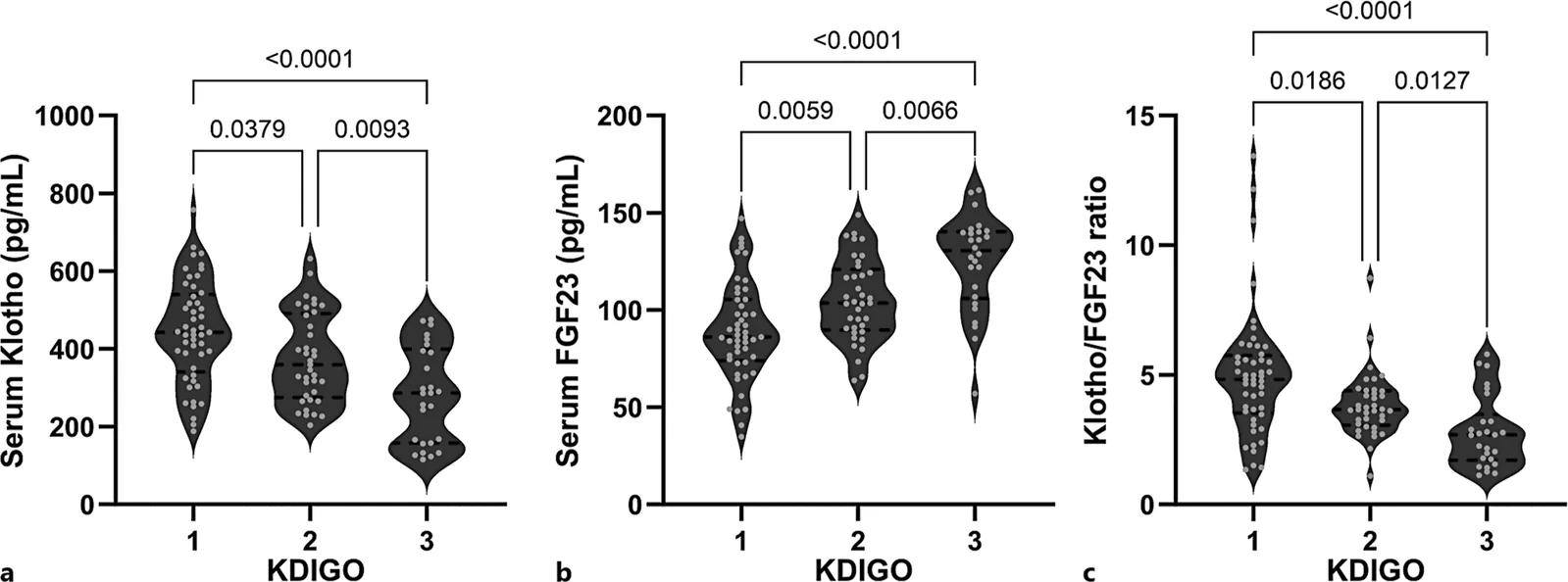
Distribution of serum Klotho, FGF23 levels and Klotho/FGF23 ratio at admission among different KDIGO stages of SA-AKI patients. **a** Klotho levels gradually decreased with the aggravation of AKI stage. **b** FGF23 levels gradually increased with the aggravation of AKI stage. **c** Klotho/FGF23 ratio gradually decreased with the aggravation of AKI stage. Data are presented as mean ± SD. *p* values were from Brown-Forsythe ANOVA test or Kruskal-Wallis *H* test.

### Association of the Admission Klotho/FGF23 Ratio with Short-Term Outcomes in SA-AKI

To examine the associations of admission Klotho, FGF23, and the Klotho/FGF23 ratio with short-term outcomes, patients were separately stratified according to the median value of each biomarker into low and high groups (*n* = 56 each), and clinical outcomes were compared (Table 4). The low-ratio group had poorer outcomes, including lower 3-month renal recovery (55.4% vs. 83.9%; *p* = 0.002), longer ICU and hospital stays (12 vs. 6 days; 21 vs. 14 days; both *p* < 0.001), higher in-hospital mortality (23.2% vs. 7.1%; *p* = 0.033), and greater 3-month progression to CKD (25.0% vs. 7.1%; *p* = 0.019). After adjustment for age ≥65 years, coronary heart disease, SOFA score, arterial lactate, and PCT, a low Klotho/FGF23 ratio remained associated with lower 3-month renal recovery (adjusted OR, 0.32; 95% CI, 0.14–0.71; *p* = 0.006), longer ICU stay (adjusted β, 0.48; 95% CI, 0.29–0.67; *p* < 0.001), longer total hospital stay (adjusted β, 0.42; 95% CI, 0.23–0.61; *p* < 0.001), higher in-hospital mortality (adjusted OR, 3.87; 95% CI, 1.12–13.35; *p* = 0.032), and greater 3-month CKD progression (adjusted OR, 4.12; 95% CI, 1.21–14.03; *p* = 0.024). Low Klotho alone was associated only with longer ICU and hospital stays, whereas high FGF23 was associated with longer ICU stay and higher in-hospital mortality. Neither Klotho nor FGF23 alone was significantly associated with all five outcomes. Collectively, a low admission Klotho/FGF23 ratio was associated with an unfavorable prognosis and showed broader outcome associations than either biomarker alone.

**Table 4.** Prognostic Outcomes of SA-AKI Patients Stratified by the Median of Klotho/FGF23 Ratio, Klotho, and FGF23

Prognostic indicators	Klotho/FGF23 ratio			Klotho alone			FGF23 alone		
	Low (*n* = 56)	High (*n* = 56)	*p* value	Low (*n* = 56)	High (*n* = 56)	*p* value	High (*n* = 56)	Low (*n* = 56)	*p* value
Renal recovery within 3 months, *n* (%)	31 (55.4)	47 (83.9)	0.002	35 (62.5)	43 (76.8)	0.097	36 (64.3)	42 (75.0)	0.153
ICU stay (days), median (IQR)	12 (8–16)	6 (4–9)	<0.001	11 (7–15)	7 (5–10)	0.003	11 (7–14)	7 (5–11)	0.015
Total hospital stay (days), median (IQR)	21 (15–28)	14 (10–18)	<0.001	19 (13–26)	16 (11–21)	0.028	20 (14–26)	15 (11–20)	0.062
In-hospital mortality, *n* (%)	13 (23.2)	4 (7.1)	0.033	11 (19.6)	6 (10.7)	0.187	12 (21.4)	5 (8.9)	0.031
CKD progression at 3 months, *n* (%)	14 (25)	4 (7.1)	0.019	12 (21.4)	6 (10.7)	0.126	11 (19.6)	7 (12.5)	0.089

Data are presented as median (interquartile range) or *n* (%). Comparisons were performed using Mann-Whitney test or Fisher’s exact test. SA-AKI, sepsis-associated acute kidney injury; CKD, chronic kidney disease.

## Discussion

In this retrospective cohort study of critically ill patients with sepsis, we found that an altered admission profile of the Klotho-FGF23 axis was associated with SA-AKI occurrence, severity, and short-term outcomes. Patients with SA-AKI exhibited a distinct admission biomarker profile characterized by reduced Klotho, elevated FGF23, and a markedly decreased Klotho/FGF23 ratio. Among these markers, the Klotho/FGF23 ratio had the highest observed AUC for SA-AKI and remained independently associated with its occurrence after adjustment for clinical covariates. Moreover, the ratio declined progressively with increasing KDIGO stage and was associated with short-term outcomes. Previous studies have examined Klotho and FGF23 in sepsis and AKI. Pei et al. [8] reported higher FGF23 levels in patients with septic AKI, although Klotho and FGF23 alone had limited discriminative value. Our study extends these findings by evaluating the Klotho/FGF23 ratio, comparing it with each biomarker alone, and examining its associations with KDIGO stage and short-term outcomes. Toro et al. [4] found that a composite index of FGF23, erythropoietin, and Klotho was associated with AKI severity, mortality, and CKD development in critically ill patients with sepsis. In contrast, our study focused specifically on the Klotho/FGF23 ratio as a measure of balance within the Klotho-FGF23 axis. Jou-Valencia et al. [10] reported reduced renal Klotho expression in patients with SA-AKI and experimental sepsis and showed protective effects of recombinant Klotho in mice. Our findings extend these observations by showing that circulating Klotho and the Klotho/FGF23 ratio were associated with SA-AKI occurrence, KDIGO stage, and short-term outcomes, although causality cannot be inferred. Christov et al. [9] summarized evidence of increased FGF23 and decreased Klotho during AKI and discussed their potential roles as biomarkers. Building on this evidence, we found that the Klotho/FGF23 ratio had the highest observed AUC among the three biomarkers. Overall, our findings provide exploratory and incremental evidence rather than the first evidence linking Klotho or FGF23 to SA-AKI. The ratio may reflect Klotho-FGF23 axis imbalance rather than changes in either biomarker alone [21]. However, the underlying mechanisms were not directly assessed, and its incremental value beyond established clinical models remains uncertain.

The Klotho/FGF23 ratio declined stepwise with increasing KDIGO stage, consistent with previous evidence that lower-ratio quartiles are associated with a higher risk of renal events [15]. This graded pattern supports an association between axis imbalance and injury burden. Although causality cannot be inferred, the observed graded association, together with reports that the ratio was independently associated with cardiovascular events and mortality in CKD [15], is consistent with an association between greater Klotho-FGF23 imbalance and more severe kidney injury. This is also consistent with CKD studies showing higher renal event risk with lower ratios, including higher risk of creatinine doubling, ≥50% eGFR decline, or end-stage kidney disease in the lowest group compared with the highest group [15].

As a kidney-derived protective protein, loss of Klotho may compromise multiple endogenous renoprotective defenses. Experimental studies show that recombinant Klotho attenuates renal inflammation and endothelial activation in sepsis models, with reduced tumor necrosis factor alpha (TNF-α) and interleukin 6 (IL-6) expression and decreased neutrophil infiltration [10, 13]. Klotho deficiency also aggravates apoptosis and endoplasmic reticulum stress, whereas Klotho supplementation limits tissue injury, in part through inhibition of p38 mitogen-activated protein kinase signaling [22]. By contrast, FGF23 rises rapidly during acute stress and has been associated with inflammatory responses. In SA-AKI, circulating FGF23 correlates positively with IL-6 [23], and elevated FGF23 has been linked to systemic inflammatory manifestations, including sepsis-related myocardial injury [24]. During sepsis, renal Klotho expression is suppressed in both human biopsies and experimental models [25], while FGF23 release increases, resulting in a “low Klotho/high FGF23” pattern [9]. The clinical consequences and direction of this relationship remain uncertain. Local and systemic inflammation may reduce renal Klotho expression through cytokine-mediated pathways [26]. Membrane-bound Klotho functions as a coreceptor for FGF23, which promotes renal phosphate excretion [27]. In AKI, reduced renal Klotho expression and decreased phosphate clearance may both contribute to elevated circulating FGF23 levels [28]. This proposed mechanism is consistent with the inverse correlation between Klotho and FGF23 and their respective associations with CRP and PCT observed in our study. Together, these findings are consistent with an association between an unfavorable Klotho-FGF23 balance and septic kidney injury. Greater renal injury may be accompanied by lower Klotho levels and higher FGF23 levels, resulting in a more pronounced axis imbalance [29]. The ratio integrates the opposing changes in Klotho and FGF23. Experimental evidence suggests that Klotho deficiency may weaken anti-inflammatory and anti-oxidative defenses [10]. FGF23 excess has been associated with inflammatory signaling [30] and may exert Klotho-independent toxicity (e.g., myocardial hypertrophy and vascular calcification) [15]. Thus, the Klotho/FGF23 ratio may provide an integrated measure of renal vulnerability during sepsis.

From a clinical perspective, early identification of patients at risk for SA-AKI remains challenging because Scr rises late and urine output is influenced by multiple non-renal factors. Measurement of the Klotho/FGF23 ratio at ICU admission showed discrimination for SA-AKI in the present cohort. Among patients with established SA-AKI, the ratio was also associated with renal recovery and short-term outcomes. However, the feasibility of routine clinical use remains uncertain. Because the ELISA assays used in this study require batch processing, their turnaround time, labor requirements, and cost may limit rapid testing in the ICU. Rapid, automated, and cost-effective assays are needed before the Klotho/FGF23 ratio can be used in routine practice.

Several limitations should be acknowledged. First, this single-center retrospective ICU cohort may have introduced selection bias. Requiring available admission serum samples and excluding patients with pre-existing chronic kidney disease may also limit generalizability. Second, SA-AKI ascertainment may have been affected by baseline creatinine definition and urine output documentation, and pre-ICU kidney injury could not be fully excluded. Third, residual confounding may remain because some relevant variables, including fluid balance, were unavailable or not included in the final models. Fourth, biomarkers were measured only at admission, precluding assessment of temporal trajectories, and assay-related variability may affect reproducibility. The turnaround time, cost, and feasibility of ELISA-based measurement for individual patients were not evaluated in this study. Finally, although bootstrap internal validation suggested limited optimism, the cut-off was derived from the same cohort and requires external validation. In addition, the incremental value of the Klotho/FGF23 ratio beyond established clinical models was not formally evaluated. Prospective multicenter studies with serial measurements and external validation are warranted.

## Conclusion

In this retrospective cohort, the admission Klotho/FGF23 ratio had the highest discrimination among the three biomarkers and remained associated with SA-AKI after adjustment. Lower ratios were also associated with higher KDIGO stage and adverse short-term outcomes. These exploratory findings require confirmation and external validation in prospective multicenter studies.

## Statement of Ethics

The study was approved by Cangzhou Central Hospital, the Approval No. was 2025-173-01, and informed written consent was waived. The study was conducted in accordance with the Declaration of Helsinki.

## Conflict of Interest Statement

The authors declare they have no conflict of interest regarding this research study.

## Funding Sources

The study was supported by Medical Science Research Project of Hebei (20261302).

## Author Contributions

Sumian Dou, Yuchao Shen, Botao Zhao, Ling Wang, Wei Zhang, Jing Wang, Chunxue Wu, Long Yang, Zhanghai Sun, and Ying Cui conducted the experiments, validation and analyzed the data, and wrote the manuscript. Sumian Dou conceived and supervised the study.

## Data Availability

The data that support the findings of this study are not publicly available due to privacy reasons but are available from Sumian Dou upon request.
